# Association of travel time with mental health service use in primary health care according to contact type — a register-based study in Kainuu, Finland

**DOI:** 10.1186/s12913-022-08815-4

**Published:** 2022-11-30

**Authors:** Tiina Lankila, Tiina Laatikainen, Katja Wikström, Miika Linna, Harri Antikainen

**Affiliations:** 1 Geography Research Unit, University of Oulu, P.O Box 8000, 90014 Oulu, Finland; 2 Department of Public Health and Social Welfare, Finnish Institute for Health and Welfare, P.O. Box 30, 00271 Helsinki, Finland; 3 Institute of Public Health and Clinical Nutrition, University of Eastern Finland, P.O. Box 1627, 70211 Kuopio, Finland; 4 Joint Municipal Authority for North Karelia Social and Health Services, (Siun Sote), Tikkamäentie 16, 80210 Joensuu, Finland; 5 Department of Health and Social Care Management, University of Eastern Finland, P.O. Box 1627, 70211 Kuopio, Finland; 6 Institute of Healthcare Engineering, management and architecture, Aalto University, Espoo, Finland

**Keywords:** Geoinformatics, Travel time, Accessibility, Mental health services, Register study, Finland

## Abstract

**Background:**

The study aim was to analyse how mental health services are used in different parts of the Kainuu region in Finland and whether travel time to primary health care services is associated with the use of different contact types (in-person visits, remote contacts, home visits).

**Methods:**

The study population included adults who had used mental health services under primary health care (*N* = 7643) between 2015 and 2019. The travel times to the nearest health centre in a municipality were estimated as the population-weighted average drive time in postal code areas. The Kruskal–Wallis test and pairwise comparisons with Dunn-Bonferroni post hoc tests were used to assess the differences in mental health service use between health centre areas. A negative binomial regression was performed for the travel time categories using different contact types of mental health service use as outcomes. Models were adjusted for gender, age, number of mental health diseases and the nearest health centre in the municipality.

**Results:**

Distance was negatively associated with mental health service use in health centre in-person visits and in home visits. In the adjusted models, there were 36% fewer in-person visits and 83% fewer home visits in distances further than 30 min, and 67% fewer home visits in a travel time distance of 15–30 min compared with 15 min travel time distance from a health centre. In the adjusted model, in remote contacts, the incidence rate ratios increased with distance, but the association was not statistically significant.

**Conclusions:**

The present study revealed significant differences in mental health service use in relation to travel time and contact type, indicating possible problems in providing services to distant areas. Long travel times can pose a barrier, especially for home care and in-person visits. Remote contacts may partly compensate for the barrier effects of long travel times in mental health services. Especially with conditions that call for the continuation and regularity of care, enabling factors, such as travel time, may be important.

**Supplementary Information:**

The online version contains supplementary material available at 10.1186/s12913-022-08815-4.

## Background

Adequate health service availability is one of the key determinants of health [1]. Long geographical distances or travel times may act as a potential barrier for the use of health services, leading to lowered service use and manifesting in poorer health outcomes and health differences between populations and areas [2, 3]. Distance is a major determinant of the *geographical accessibility* of health services, describing the geographical aspect of the fit between the health care system and its users [4]. Geographical accessibility may be the primary concern in many rural and remote areas, although other issues can also affect access to health care, namely *affordability* (client’s ability to pay for services), *availability* (needed resources to meet the client’s needs), *accommodation* (how services are organised, including opening hours) and *acceptability* (how comfortable the client and provider are with each other) of services [5–7].

A reasonable travelling time can be considered an *enabling characteristic* for health service use according to the Behavioral Model of Health Service Use [8], as it is a condition that makes the health service resources available to the individual. Other determinants of health service utilisation include *predisposing characteristics* such as age, gender or socioeconomic status that make people more likely to use certain services and *illness-level characteristics* that suggest that people may have some health-based reasons for health service use [8].

Geographic distance to health care has been identified as a significant barrier to the use of regular check-up visits and chronic care visits, especially in rural areas, whereas acute care visits appear to be less sensitive to distance [9]. Having health care provided within one’s daily activity area may be particularly important [10]. Also, fewer contacts with telephone-based out-of-hours services with increasing distance and rurality have been reported [11, 12], and fewer face-to-face consultations have been reported after the initial call [11].

While distance may affect the utilisation of all kinds of health care, there has been long-standing interest among researchers in the relationship between distance and mental health care [13], the interest can be traced back to the mid-1800s, when inverse associations between distance to the hospital and rates of admissions were first identified [14, 15]. Longer distance from service providers may aggravate the disparities in mental health service utilisation between patients of high and low socioeconomic positions [16], and it reduces the use of outpatient mental health after care following substance abuse treatment [17]. Among patients using secondary outpatient mental health services, distance seems to be negatively associated with the utilisation of community mental health centres and outpatient clinics [18]. In a recent study conducted in Switzerland, public transport travel time was also found to be negatively associated with the use of secondary outpatient services but not with inpatient ward use [19].

Regularity and continuity of care are important aspects of outpatient treatment for mental health service users [20, 21]. Especially for medical conditions that require regular contact with health care personnel, travel time may create a barrier to service use [22, 23]. Age, gender, high education, being unmarried, poor housing conditions and previous use of health services have been identified as possible predisposing factors [24–27], and having health insurance, though inconclusively, as one of the enabling factors [24, 25] for mental health service use. The number of mental health-related diagnoses constitutes the main need factor for the use of mental health services [24], others being the low level of functioning, comorbidity and perceived overall and mental health [25, 27]. In England, area-level social factors, including unemployment and deprivation, have been associated with more contacts and a higher proportion of ethnic minorities in the population, with fewer contacts in mental health services [28].

Telehealth has been viewed as an effective means of increasing mental health care access and continuity of care for people experiencing geographic, clinical or social barriers to accessing in-person care [29, 30]. In particular, telehealth services have been suggested as a promising method for delivering mental health treatment and engaging underserved populations, especially in rural settings and remote areas [31]. In a study focused on veterans with access barriers, the use of video-enabled tablets was associated with improved mental health care continuity and a lower proportion of missed or cancelled appointments when compared with a control group consisting of individuals who received mental health care from clinicians not providing telehealth care [32]. In the past two years, the COVID-19 pandemic has led to a more widespread transition from in-person to telehealth services. Although the transition has, in many cases, been an improvised response to restrictions and social-distancing precautions, there is evidence that not only are telehealth services a substitute for in-person services, but they may, in fact, contribute to an increase in the number of attended appointments and fewer cancelled appointments by minimising several logistical problems to attendance [33].

Mental health home visits and treatment have been found to be associated with both positive clinical outcomes and substantially lower costs of care compared with conventional outpatient care, especially among elderly people with depression [34, 35]. There is also evidence that mental health home visits that combine community-based and hospital-based home services can improve the stabilisation of clients’ illnesses and enhance their daily living and communication abilities [36, 37]. Despite the apparent benefits of home care services for the recipients of care, employees of home care services may have to spend a significant amount of time visiting clients. Studies have indicated that the amount of travel time may be underestimated, and the associated scarcity of time left for the actual delivery of service is a major restricting factor in providing home care services [38, 39]. Therefore, it is reasonable to assume that the geographic location of service providers, the distance between providers and recipients of mental health care services, and the available personnel exert a stronger influence on the provision of home care services than on other contact types. However, while much attention has been paid to the operational planning of service delivery [40], including route optimisation and personnel scheduling, the effect of distance on the provision of mental health home care remains a less studied area.

The purpose of our study is to investigate mental health service use in the Kainuu region in Finland from 2015 to 2019 in the adult population residing in the region, with a special focus on how mental health services are used in different parts of the region and whether travel time to primary health services acts as a potential barrier for use in different means of contacting mental health services (in-person visits, remote contacts, home visits). In this study, we focus on mental health services provided through primary health care due to the emphasis of the Finnish health care system on considering outpatient care as the primary form of treatment of patients with mental disorders [41]. Under this principle, mental health services should be organised primarily as outpatient services and clients should be supported in seeking care on their own initiative and in independent living. Mental health services provided at health centres increase the possibilities of seeking help for mental health problems, and primary care outpatient care and rehabilitation in health also costs less than hospital care.

Our study is motivated by the distinct regional differences in morbidity in Finland, including mental health and variations between rural and urban areas [42, 43]. In terms of many health indicators, northern and eastern Finland, as well as rural areas, show adverse health compared with more urbanised areas in the south and west of the country. The possible reasons for this are the lower socioeconomic status, adverse health behaviour, lack of social contacts and inadequate availability of health services in areas with higher morbidity [42]. However, the role of geographic distance and travel time to health services remains inconclusive. In a previous study focusing on the use of public primary health services by young adults in Northern Finland, distance was not found to be a major barrier to primary health care service use [44]. However, the study was based on self-reports and did not differentiate the reason for the visit, thus falling short of providing conclusive evidence on the matter.

We expect that distance may have a greater effect on in-person primary care visits (patients’ limited ability to travel) and home visits (providers’ difficulties supplying services to clients residing in remote areas) than on remote contacts. Despite a long history of studies regarding the relationship between travel distance and mental health service use, to our knowledge, the role of travel distance has not been studied explicitly with regard to the different contact types of mental health service use. This study provides information about inequalities in health service utilisation, which is essential for policy, planning and resource allocation in mental health services.

## Methods

### Study population and study area

The study population is based on health care use data from 2015 to 2019, obtained from the Finnish Care Register for Health Care [45, 46]. The register covers information on health centre and homecare service use (primary health care) and hospital outpatient and inpatient ward use (secondary care). The local authorities (municipalities or joint municipal authorities) that provide public primary health services either by themselves or through outsourcing, are obliged to provide information to the register. Health services provided in the private sector are not included in the register. In addition to health service use information (e.g. type of contact, number of contacts, diagnoses), the register provides information on the patient’s place of residence (home municipality and postal code area), age and gender. Only those patients who resided in the study region, were alive at the end of 2019 and had used mental health services in primary health care were included in the study. In the study region, 57,710 patients fulfilling the criteria had used primary health care services and the final study population, 7,643 patients had used services related to mental health between 2015 and 2019. Mental health-related service use can be identified from the register by the category of service (mental health services) and classifications for contact types.

The study region, Kainuu, with a population of 71,664 and a land area of 22,687 km2, a size comparable to Belgium [47], is located in the northern half of Finland and bordering Russia. With only 3.5 persons / km^2^, Kainuu is sparsely populated, even compared with the Finnish average of 18.2 persons / km^2^. However, there are considerable differences in population densities inside the region: more than half of the population of Kainuu live in the capital city of the region, Kajaani, with a population density of 19.9 persons / km^2 ^48. Among all regions of Finland, Kainuu had the second highest rate of mental health outpatient visits of adults per 1000 persons in 2020 [49]. In Kainuu, there have been efforts to invest in telehealth and to create a single telehealth service channel for the entire region. Indeed, Kainuu is one the region in Finland having the most remote contacts per inhabitant [50]. Municipal health services are organised in Kainuu by a joint health care authority, except for one municipality (Puolanka), which arranges municipal health services independently. There are nine primary health care facilities located in the municipality centres of the region and one central hospital located in Kajaani, the regional capital. The hospital provides 24-h unscheduled emergency, urgent and non-urgent patient care to the whole population of Kainuu.

### Health service use

Primary health care health service use in 2015–2019 was extracted from the Finnish Care Register for Health Care. Each individual contact with a health care specialist is recorded in the register, including information about the category of service, type of contact and diagnoses (ICD-10, ICPC-2) related to each contact. In this study, we separated the different types of contacts for mental health service: in-person visits to a health centre, remote contacts and home visits. In-person visits involve service in the doctor’s or health care personnel’s office, while remote contacts include service via phone, e-mail, internet, text message, video, letter or fax. Home visits include services that encompass nursing and support at home.

### Travel time to health centres

The full home addresses of the patients were not available; instead, postal code areas were used to identify the location of the patients. The travel times to the nearest health centre in a municipality were estimated as a population-weighted average drive time, based on 250 m × 250 m population grid data (Statistics Finland and Finnish Environment Institute, SYKE, 2019) and the road network (Digiroad / Finnish Transport Infrastructure Agency and Esri Finland 2019), using ArcGIS software version 10.6 (Esri, Redlands, CA). The addresses of health centres were collected from the registers of the National Institute for Health and Welfare and cross-checked against information from the municipalities’ websites. The addresses were then converted into x/y coordinates (EUREF-FIN TM35FIN) by geocoding [51]. The estimated travel time was categorised as: ≤ 15,0 min; 15,1–30,0 min; > 30,0 min. The resulting catchment areas of health centres are referred to in this study as health centre areas. Except for Kajaani, which encompasses two health centres and their catchment areas, the health centre areas correspond to the municipalities of the regions.

### Predisposing and illness level characteristics

In addition to the travel time to a health centre, other factors potentially associated with distance and the use of mental health services were examined. The gender, age and mental health diseases of patients were obtained from the Finnish Care Register for Health Care.

Mental health disorders and diseases recorded during 2015–2019 were defined from the register (both primary and secondary care) using ICD-10 and ICPC-2 codes for diagnosis. Based on the codes, the following disease groups were classified and included in the analysis: 1) psychiatric and behavioural diseases related to substance abuse (ICD-10: F10-19; ICPC-2: P16, P18-19), 2) schizophrenia and delusional diseases (ICD-10: F20-29; ICPC-2: P72, P98), 3) mood disorders (ICD-10: F30-39; ICPC-2: P73,P76), 4) neurotic, stress-related and somatoform diseases including eating disorders (ICD-10: F40-F69 (excl. F51); ICPC-2: P02, P07, P09, P74, P75, P78, P79, P80, P86, P99), and 5) sleep disorders (ICD-10: F51, G47; ICPC-2: P06). According to these groups, the total number of mental health disease groups (referred to as diseases from here on) per patient ranged from 1 to 5.

### Statistical analysis

Descriptive statistics were conducted for patient characteristics in different health centre areas (number of patients, mean and median number of mental health in-person visits, remote contacts and home visits, mean age, percentage of having mental health diseases and mean travel time) and patient and contact type characteristics in different travel time categories (number, gender, age, mental health diseases and health centre contact types for patients, and mean and median number for different contact types).

The statistical significance of differences between the distributions of different contact types of mental health service use within health centre areas was tested by the Kruskal–Wallis test, and pairwise comparisons were done with Dunn-Bonferroni post hoc tests. The average number of different contact types of primary health care mental health service use in postal code areas in Kainuu were observed on a map.

Negative binomial regression was performed for the travel time categories using different contact types of primary health care mental health service use as dependent variables. While the outcomes are count variables and they are expected to follow the Poisson distribution, in which the variance equals the mean, in our data the variance exceeded the mean, indicating over-dispersion. This was allowed for by using negative binomial regression with a dispersion parameter [52]. The crude and adjusted incidence rate ratios (IRR) and their 95% confidence intervals (95% CI) were calculated for travel time, assuming a follow-up time of five years for each patient. Models were adjusted for gender, age, number of mental health diseases, and the nearest health centre in the municipality. All the analyses were conducted using IBM SPSS Statistics version 26 (IBM corporation, 1989, 2017). There was no pre-registered analysis protocol for this register-based study. The guideline for Reporting of Observational Studies in Epidemiology (STROBE) was followed in the reporting of this study [53].

## Results

**Fig. 1:**
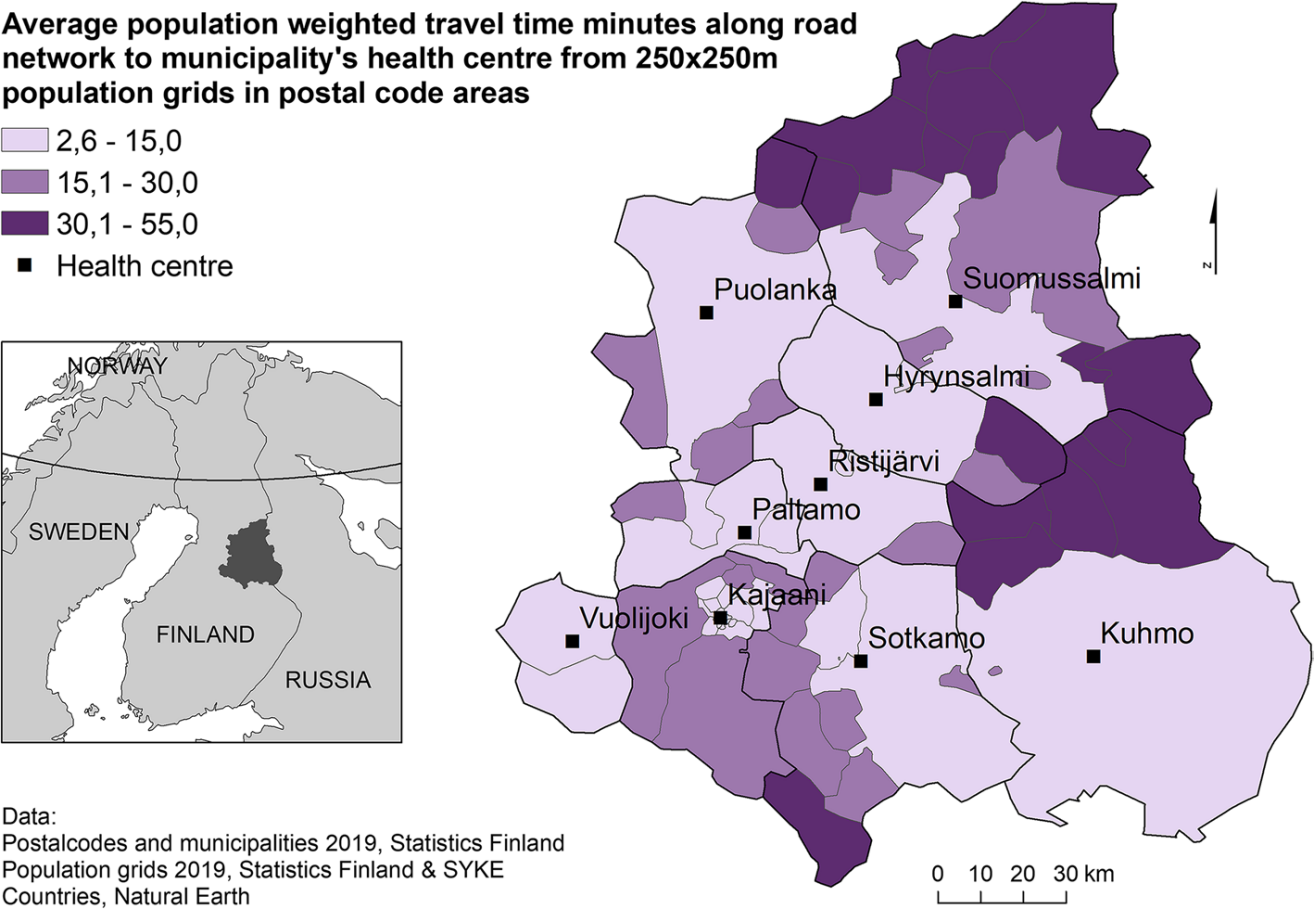
Average travel time to health centres from postal codes in health centre areas

The locations of health centres in the Kainuu region, postal code areas with their average travel time to health centres and health centre areas are shown in Fig. 1. In 28 out of 70 postal code areas, the population resides, on average, within 15 min of travel time to the health centre. The longest average travel times are found in postal code areas located in the northern and eastern parts of the region.

The study population consisted of 7643 patients, of whom 7119 had made in-person visits to a health centre, 4855 had used remote contacts and 1503 had received home visits related to mental health during 2015–2019. The mean age of patients using home services was higher (57.8 years) than that of patients making in-person visits (47.9 years) or using remote contacts (47.5 years). Also, the percentage of patients having mental health diseases was higher among patients receiving home visits (80.2) than among patients making in-person visits (71.9) or using remote contacts (76.4).

During 2015–2019, the number of patients was highest in Kajaani (3621) and smallest in the Ristijärvi (117) health centre area (Table 1). The mean number of in-person visits per patient ranged between 11.5–45.5 (median 4–7), the mean number of remote contacts between 2.3– 5.1 (median 1 in all areas), and the mean number of home visits between 0.8–6.1 (median 0 in all areas) in different health centre areas. Most patients in different health centre areas had made mental health in-person visits to a health care personnel’s office (88.4%–94.3%) and used remote contacts (52.7%–71.2%). Home visits were less common and had been received by 12.8%–32.9% of patients in different health centre areas. The mean age of the patients ranged between 43.5 years in Kajaani and 54.8 years in Puolanka, whereas the percentage of patients having mental health diseases ranged between 55.3% in Puolanka and 72.0% in Kajaani. Patients’ average travel time to a health centre, as calculated for postal code areas, varied between 5.9–12.1 min.

**Table 1:** Patient characteristics by health centre area

	Hyrynsalmi	Kajaani	Kuhmo	Paltamo	Puolanka	Ristijärvi	Sotkamo	Suomussalmi	Vuolijoki
N	251	3621	979	372	403	117	1021	712	167
In-person visits									
Mean	20,8	23,5	29,9	45,5	11,5	14,7	23,5	30,0	32,3
Median	4	7	5	5	5	3	5	6	6
%	88,4	94,1	90,3	90,6	94,3	93,2	94,2	93,1	92,2
Remote contacts									
Mean	5,1	4,4	4,0	4,4	2,91	2,3	2,5	3,7	2,8
Median	1	1	1	1		1	1	1	1
%	64,9	65,0	68,4	71,2	64,5	56,4	57,6	52,7	68,3
Home visits									
Mean	5,8	3,8	6,1	1,6	0,8	0,8	5,9	4,6	3,4
Median	0	0	0	0	0	0	0	0	0
%	31,5	13,4	32,9	18,0	13,9	12,8	20,0	31,9	29,3
Mean age	54,5	43,5	54,7	54,5	54,8	53,3	48,8	53,4	51,8
Percentage having mental health diseases^a^	67,3	72,0	71,9	70,7	55,3	61,5	70,7	65,6	65,3
Mean travel time (minutes)	8,2	5,9	9,0	8,1	8,4	7,3	9,0	12,1	8,8

^a^Psychiatric and behavioural diseases related to substance abuse; schizophrenia and delusional diseases; mood disorders; or neurotic, stress-related and somatoform diseases including eating disorders; or sleeping disorders

In-person visits: service in doctor’s or health care personnel’s office

Remote contacts: service via phone, e-mail, internet, text message, video, letter or fax

Home visits: services in patients’ place of residence

The population (*N* = 7643) consists of patients using primary health care mental health services according to the Finnish Care Register for Health Care in the Kainuu region from 2015–2019 (deceased patients are excluded)

**Fig. 2:**
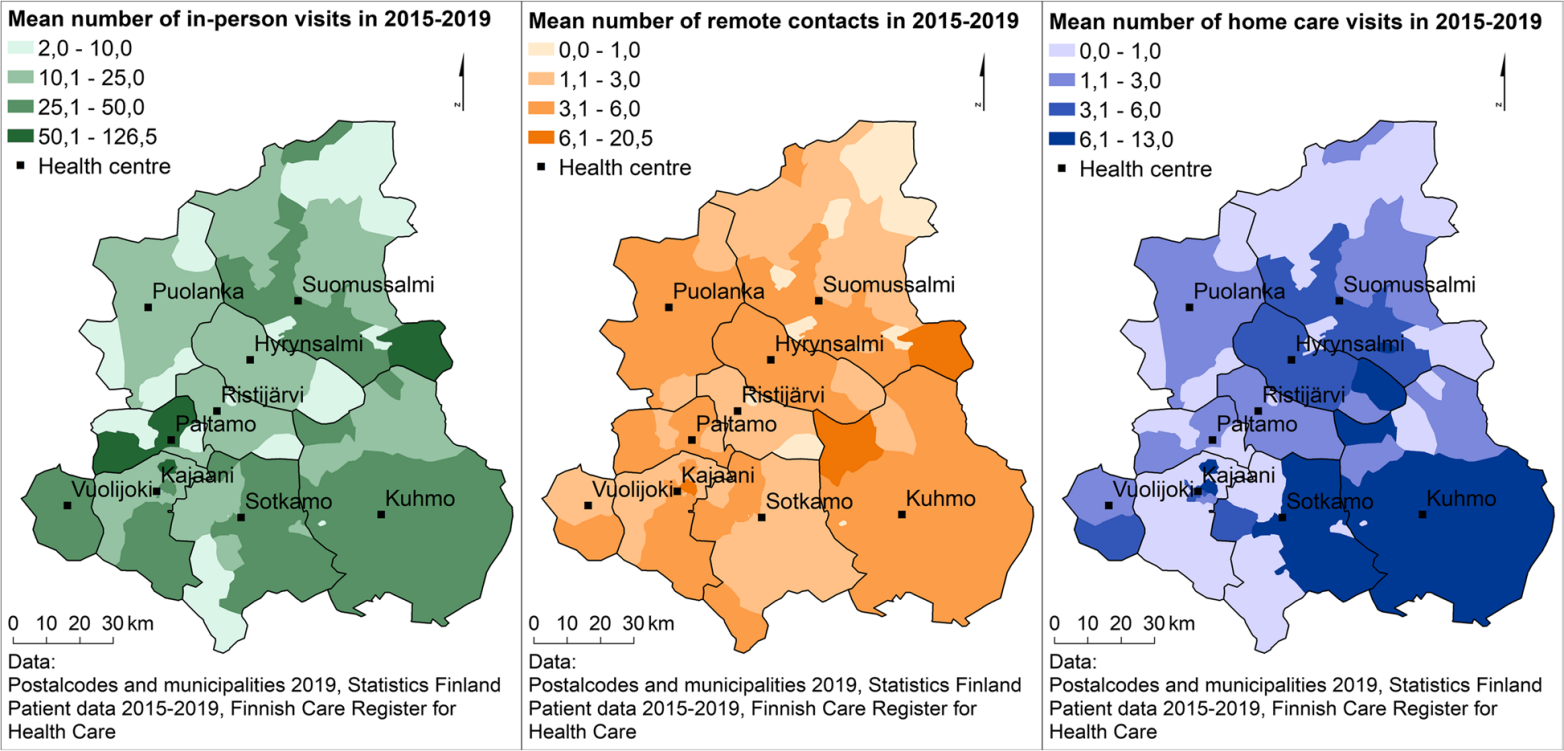
Mean number of primary health care mental health contacts per patient according to contact type

The mean number of different types of primary health care mental health contacts in postal code areas in Kainuu shows a larger variation in in-person visits than in home visits and remote contacts (Fig. 2).

According to the Kruskal–Wallis test, there were statistically significant differences in mental health in-person visits (H (df 8, N 7643) = 63,28; *p* < 0.001), remote contacts (H (df 8, N 7643) = 78,08; *p* < 0.001) and home visits (H (df 8, N 7643) = 306,19; *p* < 0.001) across health centre areas. Mean ranks and the number of significant differences between areas are shown in Table 2. The city of Kajaani differs from the other five areas in terms of in-person visits, as the average rank of in-person visits was highest in Kajaani. In remote contacts and home visits, the differences were more diverse between health centre areas: Suomussalmi, Sotkamo and Ristijärvi had the lowest and Kuhmo the highest average ranks in remote contacts, while Kajaani, Paltamo, Puolanka, Ristijärvi and Sotkamo had the lowest average ranks in home visits. More detailed pairwise comparisons between areas are available as supplementary material.

**Table 2:** Statistically significant differences in different types of contacts between health centre areas

	Hyr	Kaj	Kuh	Pal	Puo	Ris	Sot	Suo	Vuo
Contact type	Mean ranks (number of significant differences between areas*)								
ipv	3351 (4)	3985 (5)	3715 (5)	3674 (2)	3384 (4)	3257 (4)	3697 (4)	3900 (5)	3826 (3)
rc	3901 (0)	3854 (3)	4151 (4)	4040 (2)	3946 (2)	3395 (1)	3495 (4)	3481 (4)	4042 (0)
hv	4314 (5)	3583 (5)	4325 (5)	3747 (3)	3587 (4)	3552 (4)	3838 (4)	4277 (5)	4193 (3)

Hyr (Hyrynsalmi); Kaj (Kajaani); Kuh (Kuhmo); Pal (Paltamo); Puo (Puolanka); Ris (Ristijärvi); Sot (Sotkamo); Suo (Suomussalmi); Vuo (Vuolijoki)

Kruskal–Wallis test:

ipv = in-person visits: H (df 8, N 7643) = 63,28; *p* < 0,001

rc = remote contacts: H (df 8, N 7643) = 78,08; *p* < 0,001

hv = home visits: H (df 8, N 7643) = 306,19; *p* < 0,001

^*^Dunn’s pairwise comparisons with Bonferroni corrections: *p *< 0,05

The population (*N* = 7643) consists of patients using primary health care mental health services according to the Finnish Care Register for Health Care in the Kainuu region from 2015–2019 (deceased patients are excluded)

The characteristics of patients and types of mental health contacts were investigated in different travel time distances. Over 90% of patients lived in postal code areas which were on average within a 15-min drive time from the municipality’s health centre, and less than 2% lived in postal code areas which were over 30 min away from the municipality’s health centre (Table 3). There were more women than men living near the health centres, while the median age of patients was higher in distant areas. The percentage of patients having mental health-related diseases showed a U-shaped pattern, being lowest in postal code areas that were 15 to 30 min away from a health centre. The percentage of patients making in-person visits was virtually uniform across different travel distance categories, whereas the percentage of patients using remote contacts slightly decreased and patients receiving home visits slightly increased with increasing travel time. The mean number of in-person visits and home visits per patient decreased with increasing travel time, while the mean number of remote contacts per patient was quite uniform across the travel time categories.

**Table 3:** Characteristics of patients and different types of health centre contacts in different travel time distance

	Travel time minutes to nearest health centre			
	≤ 15,0	15,1–30,0	> 30,0	Total
Patients, n (%)	7076 (92,6)	429 (5,6)	138 (1,8)	7643 (100,0)
Gender				
Men	2886 (40,8)	187 (43,6)	69 (50,0)	3142 (41,1)
Women	4190 (59,2)	242(56,4)	69 (50,0)	4501 (58,9)
Age (median)	47	51	58	48
Mental health diseases^a^, n (%)	4955(70,0)	284 (66,2)	98 (71,0)	5337 (69,8)
In-person visits, n (%)	6583 (93,0)	407 (94,9)	129 (93,5)	7119 (93,1)
Remote contacts, n (%)	4528 (64,0)	244 (56,9)	83 (60,1)	4855 (63,5)
Home visits, n (%)	1398 (19,8)	75 (17,5)	30 (21,7)	1503 (19,7)
In-person visits n	181,776	9449	2218	193,443
mean (sd)	25,7 (78,4)	22,0 (50,1)	16,1 (31,1)	25,3 (76,5)
median (range)	6 (0–1589)	5 (0–500)	5 (0–248)	6 (0–1589)
Remote contacts, n	28,083	1202	487	29,772
mean (sd)	4,0 (14,2)	2,8 (6,5)	3,5 (7,6)	3,9 (13,8)
median (range)	1 (0–647)	1 (0–87)	1 (0–43)	1 (0–647)
Home visits, n	31,100	732	194	32,026
mean (sd)	4,4 (25,6)	1,7 (8,8)	1,4 (6,9)	4,2 (25,0)
median (range)	0 (0–653)	0 (0–105)	0 (0–72)	0 (0–653)

^a^Psychiatric and behavioural diseases related to substance abuse; schizophrenia and delusional diseases; mood disorders; or neurotic, stress-related and somatoform diseases including eating disorders; or sleeping disorders

In-person visits: service in doctor’s or health care personnel’s office

Remote contacts: service via phone, e-mail, internet, text message, video, letter or fax

Home visits: services in patients’ place of residence

The population (*N* = 7643) consists of patients using primary health care mental health services according to the Finnish Care Register for Health Care in the Kainuu region from 2015–2019 (deceased patients are excluded)

The incidence rate ratios (IRRs) indicate a decrease in the mental health service use with increasing travel time distance for health centre in-person visits and home visits (Table 4). There were approximately 14% fewer in-person visits to a health centre in the travel time distance category of 15–30 min and 37% fewer in-person visits to a health centre in the travel time distance category of over 30 min, compared with the 15 min travel time distance to a health centre. After adjusting for gender, age, having mental health diseases and the nearest health centre, the association became insignificant for the distance category of 15–30 min, but remained virtually unchanged (36% fewer visits) for the over 30 min distance category. As for home visits, there were 61% fewer visits in the travel time distance of 15–30 min and 68% fewer visits in the travel time distance of over 30 min compared with the 15 min travel time distance. After adjusting for gender, age, mental health diseases and the nearest health centre, associations indicated even greater differences: there were 67% and 83% fewer visits in the 15–30 min distance category and in the over 30 min distance category, respectively, compared with the 15 min distance category. In remote contacts, the IRRs indicated 29% less use in the 15–30 min travel time distance when compared with the 15 min travel time distance. After adjusting for gender, age, having mental health diseases and the nearest health centre the IRR notably increased but no statistically significant association remained with travel time distance and remote contacts.

**Table 4:** Negative binomial regression of the number of contacts to health centre for different distances

	In-person visits			
Travel time category (minutes)	Crude IRR	95% CI	Adjusted IRR^a^	95% CI
≤ 15.0	1	-	1	-
15.1–30.0	0,86	0,74–1,00	0,91	0,79–1,04
> 30.0	0,63	0,48–0,81	0,64	0,50–0,81
	Remote contacts			
Travel time category (minutes)	Crude IRR	95% CI	Adjusted IRR^a^	95% CI
≤ 15.0	1		1	
15.1–30.0	0,71	0,59–0,84	0,97	0,83–1,14
> 30.0	0,89	0,66–1,20	1,20	0,92–1,57
	Home visits			
Travel time category (minutes)	Crude IRR	95% CI	Adjusted IRR^a^	95% CI
≤ 15.0	1	-	1	-
15.1–30.0	0,39	0,25–0,61	0,33	0,22–0,51
> 30.0	0,32	0,15–0,70	0,17	0,08–0,36

IRR: incidence rate ratio; 95% CI: 95% confidence interval

^a^Adjusted for gender, age, having mental health-related diseases and the nearest health centre

In-person visits: service in doctor’s or health care personnel’s office

Remote contacts: service via phone, e-mail, internet, text message, video, letter or fax

Home visits: services in patients’ place of residence

The population (*N* = 7643) includes patients with primary health care mental health service use from the Finnish care register for health care in the Kainuu region from 2015–2019 (deceased patients are excluded)

## Discussion

To the best of our knowledge, this study is the first to examine the association of travel time with primary health care mental health service use by contact type. The study revealed significant differences in primary health care mental health service use across health centre areas and in relation to travel time in the Kainuu region of Finland. The associations varied according to how the patients contacted the services. The use of mental health services in doctors’ or health care personnel's office and home visits decreased with longer travel time, whereas remote contacts were used more uniformly across distances. Also, while all mental health contact types were most frequently used near the health centres, the percentage of patients having received home services was higher and the percentage of patients having used remote contacts was lower in distant areas. This may be an indication of difficulties in providing services to patients living in remote areas and that not all patients in distant areas may be candidates for remote services.

In particular, the distribution of in-person visits in Kajaani, the biggest population centre in Kainuu, diverged from other health centre areas, according to the statistical analysis. Kajaani differs from the surrounding municipalities in many aspects: the number of the patients is the highest, the patients are the youngest on average, the percentage of patients having mental health diseases is the highest, and the average travel time to a health centre is the shortest. These factors likely both predispose and enable patients to use in-person mental services more in Kajaani than in the surrounding areas. Also, better overall social and health service availability, including the secondary care provided in the central hospital in Kajaani, may encourage people with health problems to settle in Kajaani.

Our study concurs with other studies by suggesting that increased travel time may be associated with fewer mental health-related visits [19, 22]. Compared with patients residing near health centres in urbanised areas and population centres, patients living in sparsely populated outskirts must travel farther and may encounter other problems, such as scarcity or a complete lack of public transport [9]. What is an acceptable distance or travel time to health services from the perspective of an individual has been shown to be dependent both on contextual factors, such as the rurality or urbanity of the area, and the ability of individuals to travel [54, 55]. People living in rural areas have been found to tolerate longer travel distances better than people living in urban areas [55], and they may also be better equipped to overcome the long distances. However, a perceived lack of local services may impose feelings of insecurity on individuals needing the services even though they would have the readiness to travel to access services [54]. It is important that services are provided to patients according to their needs, as per the principles of universal health coverage and access [56].

In the case of home visits, the variation between health centre areas was more pronounced compared to other contact types. Kajaani, Paltamo, Puolanka, Ristijärvi and Sotkamo had lower average ranks in home visits than other areas. At the same time, the average age of patients was the youngest in two of these areas, Kajaani and Sotkamo. The percentage of patients having mental health diseases was the lowest in Puolanka, and the average travel times were the shortest in Kajaani and Ristijärvi, all being factors that may partly be attributable to the differences. Elderly people living alone are generally deemed prominent users of home services [57], and in this study, the patients using mental health-related home services were, on average, older than patients making either mental health in-person visits or using mental health remote contacts. Some factors on the service provider side may also account for some of the variation in service use in health centre areas in Kainuu. The availability of skilled home care personnel, long travel times and scattered residential locations of patients are things that can make service delivery difficult to manage [38, 39]. Thus, service providers may have different possibilities for offering home care to patients. Our discussions with the managers of home care in Kainuu have indicated that, rather unsurprisingly, the best home service coverage is achieved in areas in proximity to service providers. Although no definite distance thresholds for service provision exist, practice has shown that services can be provided most efficiently within about 10 kms from the facilities of the service providers, beyond which the delivery of services becomes considerably more difficult. The findings of this study also seem to strengthen this notion with regard to mental health services, as mental health-related home services were used less in the remote than in the near postal code areas. Moreover, our finding that the percentage of patients using home services is higher, but the average number of home visits is smaller in distant than in near areas, may indicate that people living far away from service providers may consist of patients who can manage their condition well, but also of people who may occasionally need home services.

The frequency of remote contacts also varied between health centre areas. Suomussalmi, Sotkamo and Ristijärvi had the lowest average rank in remote contacts and thus differed from many other areas. However, the characteristics of these areas are not fully consistent with what one might expect to decrease the use of remote contacts [58]: for example, the distance to a health centre was the longest in Suomussalmi, and the average age of patients was relatively young in Sotkamo. Kuhmo, on the other hand, had the highest average rank in remote contacts with the average age of patients and percentage of patients having mental health diseases relatively high. Still, not all studies have found an association between predisposing factors, such as gender or age, and the use of telehealth services [59]. Users of telehealth may differ distinctly from those using primary health care face-to-face services in health centres, as they may be younger, more highly educated and have a higher income [58].

The travel costs related to travel time from remote areas are often overlooked in health service use, even though they may be marked [60]. In some cases, remote contact may be a reasonable means of gaining access to health care for people living far away from service provider facilities. In our study, the percentage of those who had used remote contacts slightly decreased with distance, while the mean frequency was almost uniform. When gender, age, mental diseases and nearest health centre were considered, there were not any statistically significant associations with distance and the use of remote contacts, though the IRRS increased. In some other studies, however, telephone access to out-of-hours services has been found to decline with distance [11, 12], which does not fully agree with our study. However, in those studies, the geographical and health care setting was different from our study, and the studies specifically focused on general-practitioner services. In recent years, there have been efforts to strengthen telehealth services in the Kainuu region, which is manifested as an increase in the share of remote contacts from the year 2013 onwards [51]. Telehealth and remote contacts might be a feasible way to overcome some of the barrier effects posed by the long travel time in remote areas, although remote contacts may be appropriate for a limited type of patients and conditions only. The strengths of our study include the 5-year register-based information about actual use of mental health services in primary health care for the adult population living in the study region. Although address-level information about the residential locations of the service users was not available for privacy reasons, postal code areas and population-weighted distances based on 250 m × 250 m grid cells can be regarded as reasonably good approximations of residential locations and travel distances.

We also acknowledge certain limitations of this study. Because our study was based on register data with limited information about individual characteristics, some of the important factors associated with health service use may have been left out, such as socioeconomic status or ability to use a car [61]. In the sparsely populated areas of Kainuu, having a car may be the only feasible way to travel to health services, as the availability of public transport is very limited in the area [62]. However, people who are not able to travel to health services by themselves can use taxi transport with compensated travel costs in Finland [63, 64]. We also did not have information about the quality of mental health care in each contact, or the actual resources available in different health centres, which could have strengthened the interpretation of our findings.

In this study, we assumed that the patients used the services of the nearest health centre in their home municipality. While the majority of contacts have likely taken place in the nearest health centre or other facility in the vicinity of it in the municipal centre, it is possible that some patients may have used services in other health centres or service facilities. One factor which may obscure the associations of mental health service use and distance is that people may not always want to use the closest service provider, though once people make contact with care, they may make more frequent visits if the travel time is short. The actual premises of service use should be taken into more accurate consideration in further studies. Also, the information of patients’ residential locations was based on the last recorded postal code area from 2015 to 2019, which leaves the possibility that the patient may have also had a different home location at some point in the follow-up.

As it was not possible to account for self-selection, we are not able to know if, for example, an onset of a health problem has induced a move closer to the services, thereby increasing the use of services in postal code areas near the service facilities. People may have a propensity to move because of health-based reasons [65]. Some of the contacts that have been related to mental health service use may have been recorded as other types of visits, such as substance abuse-related visits, meaning that the mental health-related visits may be under-represented in some cases. Also, those having mental health problems, but not having diagnosis and never seeking services, are missing from the data. On the other hand, people who have visited health services more frequently are more likely to have had more disorders diagnosed, though the use of disease groups rather than individual diagnoses as a confounder decreases the impact of this. It should also be noted that the use of mental health services should not be taken as an indicator of the need for mental health care in different areas, because several factors may either promote or hinder the user of services [2]. The generalisability of the findings in this study may be partly limited to other similar, sparsely populated areas with publicly funded health care systems.

While previous research suggests that the geographical accessibility of Finnish primary health care is mostly good [62], the ageing population and lack of skilled personnel in certain areas may deter the situation in the coming years. It is important to make potential inequalities in health service use and provision visible so that they can be considered when planning and providing health services. According to Anderson & Newman [8], it may be more feasible to bring about a change through the enabling (e.g. travel time to service) than the predisposing variables (e.g. age, gender). Earlier policy decisions concerning service provision may have been made with very limited information, signifying that studies calling attention to the importance of different societal and individual determinants of health and health service use are valuable.

## Conclusion

The present study revealed significant differences in primary health care mental health service use in relation to travel time and according to how services were contacted in the Kainuu region, Finland. The mental health in-person visits and home visits decreased with longer travel time, whereas remote contacts were used more uniformly across distances. Policy decisions concerning service provision should be informed about the importance of the individual, societal and provider-side determinants of health and health service use and possible inequalities related to these factors. Especially with conditions that call for the continuation and regularity of care, enabling factors, such as travel time, may be crucial. Providing services remotely may partly compensate for the barrier effects of long travel times in mental health services. Because of the study context, the results may be best applicable to sparsely populated areas with publicly funded health care systems.

## Supplementary Information

**Additional file 1.** Pairwise comparisons of different types of contacts between health centre areas.

## Data Availability

The data that support the findings of this study are available from the Finnish Institute for Health and Welfare, but restrictions apply to the availability of these data, which were used under license for the current study, and so are not publicly available due to Finnish data protection legislation. According to the legislation, register authorities give permissions to use register data including sensitive individual information (e.g., health data) to study specified research questions to named individuals who have signed a pledge of secrecy and they are not permitted to forward it to other researchers. Other researchers can apply for the data from the Health and Social Data Permit Authority Findata. Findata handles the data permit applications concerning Finnish Institute for Health and Welfare registers (the Care Register for Health Care in the current study), see https://www.findata.fi/en/services/data-requests/. In issues related to the data availability the corresponding author Tiina Lankila can be contacted.

## Abbreviations

**N**: Number
**COVID-19**: Coronavirus disease 2019
**ICD-10**: International Classification of Diseases 10th Revision
**ICPC-2**: International Classification of Primary Care
**IRR**: Incidence rate ratio
**95% CI**: 95% Confidence interval
**H**: Kruskal–Wallis Test
**df**: Degree of freedom
**p**: Probability
**ipv**: In-person visit
**rc**: Remote contact
**hv**: Home visit
**sd**: Standard deviation
