# Supplementary material for: Association of travel time with mental health service use in primary health care according to contact type — a register-based study in Kainuu, Finland
Source: BMC Health Serv Res. 2022 Nov 30;22:1458. doi: 10.1186/s12913-022-08815-4 (PMC9713086; doi:10.1186/s12913-022-08815-4)
Supplement: Supplementary file 1 — Additional file 1. Pairwise comparisons of different types of contacts between health centre areas. [file 12913_2022_8815_MOESM1_ESM.docx]

| Pairwise comparisons of different types of contacts between health centre areas. | | | | | | | | | | |
| --- | --- | --- | --- | --- | --- | --- | --- | --- | --- | --- |
| Mean  ranks | Area | Hyr | Kaj | Kuh | Pal | Puo | Ris | Sot | Suo | Vuo |
| ipv 3753  rc 4338  hv 4765 | Hyr | - | ipv***  hv*** |  | hv*** | ipv***  hv*** | ipv***  hv*** | ipv***  hv*** |  |  |
| ipv 4395  rc 4264  hv 3944 | Kaj | ipv***  hv*** | - | ipv***  rc**  hv*** |  |  |  | ipv***  rc***  hv*** | ipv***  rc**  hv*** | ipv***  hv*** |
| ipv 4109  rc 4465  hv 4763 | Kuh |  | ipv***  rc**  hv*** | - | ipv***  hv*** | ipv***  hv*** | ipv***  rc*  hv*** | ipv***  rc*** | rc*** |  |
| ipv 4142  rc 4264  hv 4099 | Pal | hv*** |  | ipv***  hv*** | - |  |  | rc** | ipv***  rc**  hv*** |  |
| ipv 3765  rc 4422  hv 4009 | Puo | ipv***  hv** |  | ipv***  hv*** |  | - |  | rc* | ipv***  rc*  hv*** | ipv***  hv** |
| ipv 3745  rc 3535  hv 3862 | Ris | ipv***  hv*** |  | ipv***  rc*  hv*** |  |  | - |  | ipv***  hv*** | ipv*  hv* |
| ipv 4070  rc 3927  hv 4230 | Sot | ipv***  hv*** | ipv***  rc***  hv*** | ipv***  rc***  hv*** | rc** | rc* |  | - | ipv***  hv*** |  |
| ipv 4221  rc 3971  hv 4809 | Suo |  | ipv***  rc**  hv*** | rc*** | ipv***  rc**  hv*** | ipv***  rc*  hv*** | ipv***  hv*** | ipv***  hv*** | - |  |
| ipv 4172  rc 4347  hv 4564 | Vuo |  | ipv***  hv*** |  |  | ipv***  hv** | ipv*  hv* |  |  | - |
| Hyr (Hyrynsalmi); Kaj (Kajaani); Kuh (Kuhmo); Pal (Paltamo); Puo (Puolanka); Ris (Ristijärvi); Sot (Sotkamo); Suo (Suomussalmi); Vuo (Vuolijoki).  Kruskal–Wallis test:  ipv = in-person visits: H (df 8, N 7643) = 63,28; p < 0,001  rc = remote contacts: H (df 8, N 7643) = 78,08; p < 0,001  hv = home visits: H (df 8, N 7643) = 306,19; p < 0,001  Dunn’s pairwise comparisons with Bonferroni corrections: * <0,05; ** <0,01; *** <0,001  The population (N = 7643) consists of patients using primary health care mental health services according to the Finnish Care Register for Health Care in the Kainuu region from 2015–2019 (deceased patients are excluded). | | | | | | | | | | |
